# Anti-*c-myc* RNAi-Based Onconanotherapeutics

**DOI:** 10.3390/biomedicines8120612

**Published:** 2020-12-15

**Authors:** Saffiya Habib, Mario Ariatti, Moganavelli Singh

**Affiliations:** Nano-Gene and Drug Delivery Group, Discipline of Biochemistry, University of KwaZulu-Natal, Private Bag, Durban X54001, South Africa; saffiya.habib@gmail.com (S.H.); ariattim@ukzn.ac.za (M.A.)

**Keywords:** *c-myc*, RNA interference, siRNA, oncogene, gene silencing, expression, nanosystems

## Abstract

Overexpression of the *c-myc* proto-oncogene features prominently in most human cancers. Early studies established that inhibiting the expression of oncogenic *c-myc*, produced potent anti-cancer effects. This gave rise to the notion that an appropriate *c-myc* silencing agent might provide a broadly applicable and more effective form of cancer treatment than is currently available. The endogenous mechanism of RNA interference (RNAi), through which small RNA molecules induce gene silencing by binding to complementary mRNA transcripts, represents an attractive avenue for *c-myc* inhibition. However, the development of a clinically viable, anti-*c-myc* RNAi-based platform is largely dependent upon the design of an appropriate carrier of the effector nucleic acids. To date, organic and inorganic nanoparticles were assessed both in vitro and in vivo, as carriers of small interfering RNA (siRNA), DICER-substrate siRNA (DsiRNA), and short hairpin RNA (shRNA) expression plasmids, directed against the *c-myc* oncogene. We review here the various anti-*c-myc* RNAi-based nanosystems that have come to the fore, especially between 2005 and 2020.

## 1. Introduction

The *c-myc* gene encodes a nuclear phosphoprotein that is widely recognized for its role as a transcription factor. The c-Myc protein is believed to participate in the regulation of 10–15% of all genes [1]. These include genes involved in cell cycle progression [2,3], metabolism [4], cell growth [5,6,7], differentiation [8], adhesion [9], and apoptosis [10]. 

**Figure 1:**
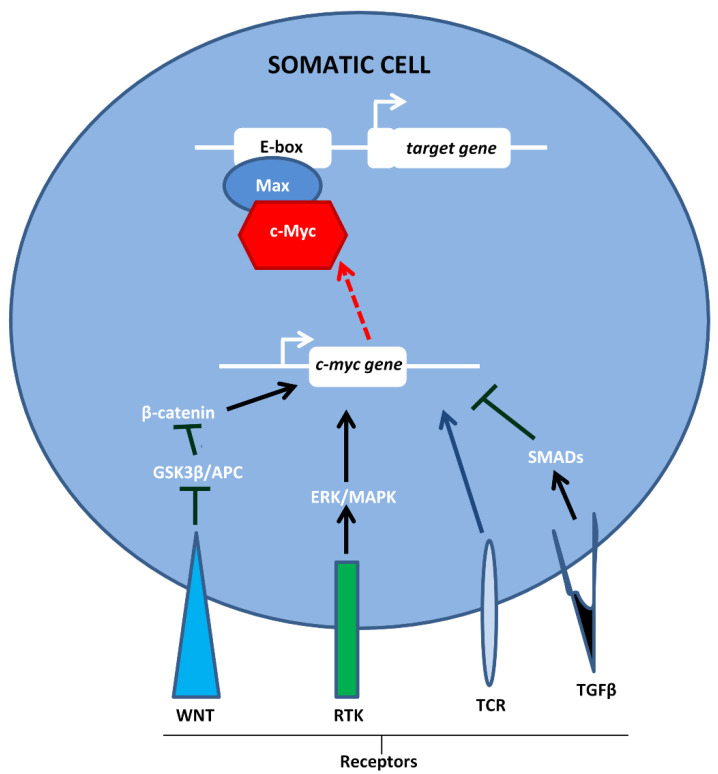
Transcription of the *c-myc* gene in normal cells occurs in response to signals from membrane-anchored receptors. White arrows show the direction of transcription (adapted from [18]).

Due to its function in regulating essential cellular functions, expression of the *c-myc* gene and activity of the c-Myc protein is, under normal circumstances, tightly controlled. Control points include the transcriptional regulation of the *c-myc* gene itself [11,12], the activity of translation initiation factor eIF4E, which ensures that only faithful *c-myc* mRNA transcripts are exported to the cytoplasm [13]; the short half-life of *c-myc* mRNA [14], post-translational modifications such as phosphorylation, acetylation, and ubiquitinylation [15]; and proteins which either directly interact with c-Myc [16] or influence dimerization with its obligate partner protein, Max [17]. As shown in Figure 1, *c-myc* functions in response to signals from several ligand membrane receptor complexes, which cause either positive or negative regulation. When associated with Max, c-Myc binds to DNA E-boxes and this, in turn, regulates the transcription of its target genes [18].

Abnormal *c-myc* expression can occur due to genetic events that include translocations [19], rearrangements [20], and amplification [21], as well as flaws in the pathways implicated in the regulation of this gene or the protein that it encodes [22]. Research carried out in the 1980s showed an association between the deregulated expression of *c-myc* and tumorigenesis [23,24]. Further work showed that abnormal *c-myc* expression causes neoplastic changes, by eliminating check-points in the cell cycle [25,26], prompting genomic instability [27], and through association with other oncogenes [28,29]. In fact, tumor cells often rely on *c-myc* expression for the maintenance of the cancerous state. This phenomenon, known as the oncogene addiction, was emphasized by studies that showed that *c-myc* inactivation caused tumor regression in transgenic mice [30], by inhibiting the cellular proliferation and inducing senescence or apoptosis and differentiation [31]. Moreover, the effects of systemic *c-myc* inhibition were found to be mild in normal tissues, and were well tolerated over time [32]. These findings, together with an estimation that *c-myc* is deregulated in up to 70% of human cancers [18], making it the most frequently altered oncogene, motivate strongly for the therapeutic value of inhibiting oncogenic *c-myc*.

In theory, the oncogenic activity of *c-myc* can be eliminated by inhibiting the expression of the activated gene, inhibiting inter-protein associations that are critical for c-Myc function, or by disrupting pathways that support *c-myc* deregulation in cancer cells. This provided a basis for the design and evaluation of several potential anti-cancer strategies. The antisense oligonucleotides were featured in some of the earliest reports of *c-myc* inhibition [33,34,35]. The application of antisense technology to c-Myc inhibition expanded with nucleotide modifications designed to confer greater stability and specificity [36,37]. However, Nobel Prize-winning work that described an endogenous gene silencing mechanism, known as RNA interference (RNAi) [38], presented further possibilities.

Short RNA duplexes of 19–21 base pairs with 2 nucleotide 3′ overhangs, known as small interfering RNA (siRNA), are the key mediators of this pathway. siRNA associates with a network of cytoplasmic proteins to form the RNA-induced silencing complex (RISC), through which it guides the degradation of mRNA, bearing a complementary sequence [39]. Short dsRNA molecules, lacking the dinucleotide overhangs that typify siRNA, termed DICER-substrate siRNA (DsiRNA), can also induce RNAi, with a reportedly higher efficiency. These are processed by the enzyme DICER into siRNA molecules that associate with the RNAi machinery [40]. DNA-directed RNAi, a strategy that generates specific siRNA molecules in vivo, is a useful gene-silencing tool [41]. This involves the construction of a RNA pol-driven plasmid expression vector, into which an antigene sequence of at least 19 nucleotides is inserted, together with appropriate termination signals. When introduced into cells, the antigene sequence is transcribed in the nucleus as a stem-loop structure, which is essentially 2 complementary sequences, 19–22 ribonucleotides in length, linked by a short loop of 4–11 ribonucleotides. This is known as short hairpin RNA (shRNA). The shRNA is exported to the cytoplasm where it is processed into siRNA by DICER [42]. In theory, effective silencing of *c-myc*, or any oncogene, might be achieved using endogenous cellular machinery, provided that the appropriately designed effector nucleic acid is successfully introduced. However, several factors militate against the success of naked nucleic acids in vivo. Naked nucleic acids are highly susceptible to serum nucleases [43] and are rapidly cleared by the kidneys [44]. However, studies have reported that chemical modifications such 2′-*O*-methylation of the guide strand [45], and the use of a passenger 3′ 19ntDNA/siRNA construct (previously referred to as “crook siRNA”), endowed the nucleic acid with nuclease resistance [46].

Furthermore, the size and net negative charge prevent passage across biological membranes [47]. Much effort was focused on the design of delivery agents that would mask its negative charge, protect its integrity, prevent its early removal from the body and facilitate cellular entry. In this regard, nano delivery systems received considerable attention, many of which are based on the principle that nucleic acids can electrostatically associate with positively charged agents [48]. This review discusses the potential application of anti-*c-myc* RNAi nanosystems in cancer treatment.

## 2. Anti-*c-myc*-siRNA

### 2.1. Lipid-Based Nanosystems

**Figure 2:**
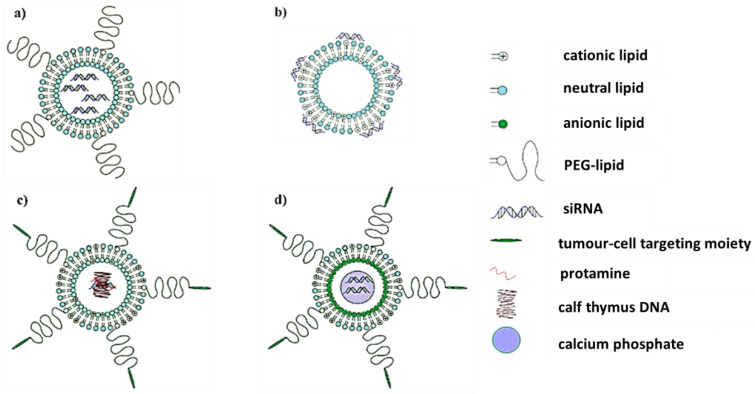
Lipid-based delivery agents for anti-*c-myc* siRNA (**a**) pegylated, neutral liposome, (**b**) cationic liposome, (**c**) liposome-polycation-DNA (LPD) nanoparticle, and (**d**) lipid calcium phosphate (LCP) nanoparticle. Images were created using DesignSpark Mechanical 2.0 software.

The liposome is arguably the least complicated lipid-based delivery agent—the simplest of which is a self-assembled phospholipid bilayer that encircles an aqueous core in which a variety of molecules might be entrapped [49]. It is this carrying capability that was exploited for the delivery of several therapeutically important molecules, including siRNA. A neutral liposome composed of dioleoylphosphatidylcholine (DOPC), cholesterol (Chol), and distearoylphosphatidylethanolamine- poly(ethylene glycol) (DSPE-PEG) was used to encapsulate and deliver anti-*c-myc* siRNA in vivo [50]. Pegylation, the introduction of the PEG polymer, served to create a hydration shell around the liposome that sterically inhibits adverse interparticle associations that reduce nanoparticle longevity in the body [51]. Systemic administration of the DOPC/Chol/DSPE-PEG/siRNA complex reduced the growth of ovarian cancer xenograft tumors and did not inhibit the growth of cells with low *c-myc* expression [41]. Anti-*c-myc* siRNA delivered via pegylated DOPC liposomes also showed promise in the treatment of cisplatin-resistant ovarian tumors [52]. Figure 2 provides a representation of some lipid-based delivery systems for anti*-c-myc*-siRNA.

Felgner et al. [53] first reported that the hydration of a mixture containing a synthetic cationic lipid and zwitterionic phospholipid create vesicles that bear a net positive charge, and paved the way for the use of cationic liposomes in nucleic acid delivery. Unlike neutral liposomes in which the siRNA must be encapsulated, cationic liposomes electrostatically associate with siRNA to form nanostructures, known as lipoplexes [54]. Early experiments involved the use of a commercially available cationic liposomal reagent, LipofectamineTM 2000, to demonstrate the therapeutic value of siRNA-mediated c-myc inhibition in human colon cancer [55]. Later, liposomes prepared from equimolar quantities of the cationic lipid *N*,*N*-dimethylaminopropylamidosuccinyl- cholesterylformylhydrazide (MS09), and Chol proved to be simple, but effective anti-*c-myc* agents, which elicited apoptotic cancer cell death and loss of migratory potential in colorectal and breast carcinoma cell lines [56].

Besides being limited to use in cationic liposome formulations, cationic lipids contributed to the development of more elaborate lipid nanoparticles. For example, Chen et al. [57] used a traditional cationic liposome made up of 1,2-dioleoyl-3-trimethylammonium-propane (DOTAP) and Chol, to envelope a core of protamine-bound anti-*c-myc* siRNA and calf thymus DNA (Figure 2). This is known as a liposome-polycation-DNA (LPD) nanoparticle. Surface modifications included post-inserted PEG chains for steric stabilization and a peptide directed to aminopeptidase N, that is overexpressed by cancer cells. Effective siRNA delivery, *c-myc* inhibition, and tumor cell apoptosis were noted after these nanoparticles were intravenously administered in a xenograft model. Co-formulation of the anti-cancer drug doxorubicin with siRNA in targeted LPD nanoparticles further improved treatment efficacy [57]. Following the concept of stabilized core/shell lipid nano-assemblies, Zhang et al. [58] used a DOTAP/Chol/PEG formulation as the outer coating of a calcium phosphate core containing anti-*c-myc* siRNA (Figure 2). The resulting lipid calcium phosphate (LCP) nanoparticle was directed to sigma receptor-positive tumor cells by attachment of anisamide to the distal ends of PEG chains. Similar to the findings of Chen et al. [57], co-encapsulation of anti-*c-myc* siRNA and a chemotherapeutic agent, in this case, gemcitabine, gave a more pronounced anti-cancer effect.

Physical agents might prove useful in promoting the deposition of systemically introduced liposomal anti-*c-myc* siRNA nanoparticles in tumors. A tumor-targeted formulation of 3β[*N*-(*N*′,*N*′-dimethylaminoethane)-carbamoyl] cholesterol (DC-Chol), Chol, and DSPE-PEG with a photolabile-caged cell-penetrating peptide was used to deliver anti-*c-myc* siRNA. The application of near-infrared light at the tumor site, activated the cell-penetrating ability of the peptide [59]. Liposomes were also used as ultrasound cavitation agents for site-specific release of anti-*c-myc* siRNA conjugated to a cell-penetrating peptide [60]. In both instances, treatment delayed tumor progression in fibrosarcoma xenograft models.

Anti-*c-myc* siRNA was included in multi-targeted anti-cancer strategies, which involve the combined delivery of siRNAs against several genes implicated in cancer. A mixture of siRNAs against *c-myc*, *MDM2*, and *VEGF* was shown to inhibit tumor growth more effectively than the individual siRNAs [61]. Li et al. [62] co-encapsulated siRNA molecules against the same targets in a pegylated LPD nanocarrier, for systemic administration in a murine model of metastatic lung cancer. This treatment simultaneously silenced all three genes in cancerous tissue, reduced metastasis by approximately 80%, and extended survival time, with minimal toxicity. Similar results were obtained when siRNAs against the aforementioned oncogenes were pooled in pegylated LCP nanoparticles [63]. Later, a mechanistic study showed that this system impaired the growth of tumors in mice, by simultaneously inhibiting cell proliferation and angiogenesis [64].

Besides delivery via synthetic lipid vesicles, it is worth mentioning that siRNA can also be loaded in exosomes. Exosomes are vesicles that are naturally released by cells for the purposes of intercellular communication and represent an emerging nanocarrier system for a variety of medically relevant molecules [65]. The potential for exosome-mediated anti-*c-myc* siRNA delivery was demonstrated by Lunavat et al. [66] in vitro. 

### 2.2. Miscellaneous Organic Nanosystems

Other organic anti-*c-myc* nano delivery systems reported are often complex polymer- and peptide-based nanocomposites. Folate-targeted, pegylated chitosan nanoparticles were used to encapsulate anti-*c-myc* siRNA associated with packaging RNA, to give a dual-targeting anti-tumor system that improved cellular uptake, gene silencing, and cancer cell death [67]. As a further example, Raichur et al. [68] used a layer-by-layer approach to associate anti-*c-myc* siRNA with poly(lactic-co-glycolic acid) hollow nanoparticles. In vitro experiments showed that the nanoparticles were taken up by aggressive cancer cells and reduced *c-myc* expression with loss of cell viability. More recently, Misra et al. [69] achieved approximately 90% growth inhibition in human melanoma with a nano assembly of palmitoyl-bioconjugated acetyl coenzyme-A termed “siNozyme”, which co-delivered anti-*c-myc* siRNA and the chemotherapeutic agent, amonafide. 

Anti-*c-myc* siRNA is associated with cell-penetrating peptides (CPP), the simplest of which is an epidermal growth factor receptor-targeted fusion peptide, SPACE–EGF, for topical application to skin cancers [70]. A more complex peptide assembly that contained cationic peptides for siRNA-binding, pH-sensitive peptides for endosomal escape, and a tumor-targeting motif was used for the simultaneous delivery of siRNA against *c-myc* and *Stat3*. This system markedly reduced anchorage-independent growth in recalcitrant breast cancer cells [71]. An elaborate system consisting of a disulfide linked anti-*c-myc* siRNA-CPP encapsulated by a thermosensitive liposome, decorated with a tumor-targeting peptide motif showed effective *c-myc* silencing and antitumor activity in a fibrosarcoma xenograft model [72]. In a related study, an anti-*c-myc* siRNA-CPP conjugate contained within thermal and magnetic dual-responsive liposomes gave encouraging results in a murine breast cancer model [73].

### 2.3. Inorganic Nanosystems

The use of inorganic nanoparticles in siRNA delivery was explored in recent years. These are often modified with organic components to improve surface properties and reduce toxicity [74,75,76,77,78]. Attachment of siRNA involves either covalent conjugation or electrostatic association with positively charged groups introduced on the surface of the nanoparticle [79,80]. Anti-*c-myc* siRNA carried by PEG- [81], poly(ethylene imine)- [82], and chitosan- [83] functionalized gold nanoparticles was shown to reduce *c-myc* expression in human cervical, liver, and breast cancer cell lines, respectively. In separate in vivo experiments, gold nanoparticles modified with cationic [84] and anionic polymer shells [85], glucose residues [86], and an RGD tumor-targeting peptide [87], delivered anti-*c-myc* siRNA and suppressed the growth of lung tumors.

Nanoparticles based on selenium [77,88] and graphene oxide [82] was also introduced as potential carriers of anti-*c-myc* siRNA. Huang et al. [88] modified a doxorubicin selenium core with RGD-linked polyamidoamine for cancer cell-specific combination therapy, using anti-*c-myc* siRNA. The resultant nanostructure was serum-stable, successfully penetrated the blood–brain barrier and inhibited the growth of glioblastoma spheroids in vitro. In the same year, Imani et al. [89] showed that nano-graphene oxide with PEG and octaarginine conjugation effectively delivered anti-*c-myc* siRNA to human breast cancer cell lines, due to its superior stability and cell-penetrating ability. Some proof of principle studies using nanoparticles such as gold [78], selenium [77], and hydrotalcites [90], showed the potential for the delivery of anti-*Luc*-siRNA, paving the way for the delivery of other therapeutic siRNA molecules, including anti-*c-myc* siRNA.

## 3. DsiRNA

Like conventional siRNA, DsiRNA requires a vehicle for successful entry. Of significance to this discussion is the fact that pharmaceutical company, Dicerna, reported on a DsiRNA specific for the c*-myc* oncogene, DCR-MYC, delivered using a proprietary EnCore^™^ lipid nanoparticle. DCR-MYC in this delivery platform is the first, and only anti-*c-myc* RNAi system, to date, to have reached clinical trials [91]. Although the outcome of the initial trial was encouraging, a subsequent trial showed an unsatisfactory knockdown efficiency and its development was discontinued [92].

## 4. Anti-*c-myc*-shRNA

Most experiments with anti-*c-myc* shRNA plasmids involved their introduction into cells in culture, with the aid of commercial cationic lipid transfection reagents. In one such study, plasmid-driven anti-*c-myc* shRNA silenced *c-myc* expression by as much as 80%, reduced the colony-forming ability, and promoted apoptosis in MCF-7 breast cancer cells [93]. A similar plasmid system impaired proliferation, invasion, and motility in the hepatocellular carcinoma cell line, HepG2 [94]. The transfection of colon cancer cells with anti-*c-myc* shRNA plasmids not only reduced *c-myc* expression, but also that of the human telomerase reverse transcriptase gene (*hTERT*), which is under the transcriptional regulation of *c-myc*, and also contributes towards carcinogenesis, when abnormally expressed [95].

As with siRNA, the effect of multigene silencing using shRNA expression plasmids was also explored. A single plasmid was engineered to direct the transcription of shRNAs against *c-myc*, *VEGF*, *hTERT*, and *BIRC5*, which encodes *Survivin*. This produced a more effective anti-cancer effect than shRNA plasmids targeting individual oncogenes [96]. Similarly, Tai et al. [97] observed a synergistic anti-cancer effect, in colon cancer cells, when the cells were co-transfected with two shRNA plasmids, each separately targeting *c-myc* and *VEGF*. The field of anti-*c-myc* RNAi also benefitted from advances in the design of shRNA-encoding vectors. Recently Cheng et al. [98] used branched PCR technology to introduce a multisite-targeting c-Myc shRNA array into DNA nanovectors that reduced cellular *c-myc* mRNA levels by approximately 98%.

Thus far, only one in vivo experiment with anti-*c-myc* shRNA was reported. In this study, a poly(ethylene imine)-grafted polyglycidal methacrylate nanoparticle was used as a carrier of the shRNA expression vector. Anti-*c-myc* shRNA delivered in this manner suppressed tumor growth in murine models of breast and colon cancer [99].

Table 1 provides a summary of the anti-*c-myc* RNAi-based systems developed to date. The systems discussed were selectively used for the delivery of anti-*c-myc* RNAi molecules. Nano-delivery systems such as mesoporous silica nanoparticles [100,101] and magnetic nanoparticles [102,103] are not discussed in this review, but showed potential in drug delivery, which can be considered for as future carriers of siRNA, shRNA, or DsiRNA.

## 5. Conclusions

Anti-*c-myc* RNAi-based nanosystems have, in many instances, induced potent anti-cancer effects in vitro and in vivo. To date, only DsiRNA was evaluated as an alternative cancer treatment in clinical trials but did not progress further. Several groups and companies are pursuing the idea of inhibiting *c-myc* at the level of translation as a means of designing a clinically viable anti-*c-myc* agent. Hence, RNAi-based strategies are currently significant [93]. Although longer lasting oncogene inhibition can be achieved with DNA-directed RNAi [104], mature siRNA molecules are easily synthesized and pose fewer delivery concerns, as they are of lower molecular weight and do not require genome integration [48,105]. Hence, siRNA is considered more suitable for therapeutic use.

While gene expression might be interrupted by other means such as the restriction enzyme-based system, CRISPR/Cas9, RNAi is most likely the better strategy for *c-myc* inhibition. Given that the RNAi apparatus is present in all mammalian somatic cells, no prior genetic manipulation of the diseased cell line is needed [106]. This is a massive advantage because simple, transient transfection with anti-*c-myc* siRNA is sufficient to achieve anticancer activity [106,107,108]. Moreover, since RNAi occurs in the cytoplasm, there are no issues with chromatin accessibility, which can perturb gene-editing attempts with CRISPR/Cas9 technology. It is worth mentioning at this point that RNAi is not without its drawbacks, notably the occurrence of off-target effects. However, these are relatively easily attenuated by careful optimization of the design and dose of anti-*c-myc* siRNA molecules [106].

Research to date has emphasized that the development of a suitable anti-c-myc agent is largely dependent upon the design of an appropriate nano delivery system. Great strides were made since the first anti-*c-myc* oligomers were introduced in nanoparticle form [109,110,111]. siRNA, which functions catalytically and non-stoichiometrically, has surpassed the potency of the antisense oligomers. In recent years, nanosystems were used to deliver small molecule inhibitors of c-Myc-Max dimerization in prodrug form. Initially plagued by issues such as poor bioavailability, low solubility, rapid metabolism, and low potency; their incorporation into nanoparticles showed promise [112,113,114]. However, anti-*c-myc* RNAi nanotechnology is, at present, a more developed field, and presents a large body of knowledge upon which to improve. 

Of all nucleic acid carriers explored thus far, the most significant development were made in the field of lipid-based delivery. Inorganic nanoplatforms, an emerging field, served to solidify the notion that anti-*c-myc* RNAi is a potent anti-cancer instrument. However, with systemic administration, RNAi nanoparticles still have significant challenges to overcome. Their entry into clinical trials might highlight difficulties that include poor retention time in the body and target-site penetration [115]. To this end, polymer and ligand-targeting modifications are common features of several anti-*c-myc* RNAi nanosystems. Such advances in nanoparticle design and expanding test systems for the newly developed nanoparticles represent additional avenues of research in this field. Hence, the advent of a clinically viable anti-*c-myc* RNAi-based anti-neoplastic agent is eagerly awaited.

## Figures and Tables

**Table 1:** Summary of anti-*c-myc* RNAi-based nanosystems developed to date. Symbols **✓** = present/yes; **✗** = absent/no.

Nucleic Acid	Carrier	Polymer Modification (✓/✗)	Ligand-Targeting Modification (✓/✗)	Disease State	Test System	Advantages	Disadvantages	Clinical Trial (✓/✗)	Reference
siRNA	neutral liposome	✓	✗	Ovarian cancer	Xenograft tumors	High encapsulation efficiency Good biocompatibility Low toxicity Weak immunogenicity	Low transfection efficiency High production cost	✗	[50,52]
	cationic liposome	✗	✗	Colon cancer Breast cancer	HT-29 cells MCF-7 cells	High encapsulation efficiency Good biocompatibility Low toxicity Weak immunogenicity	Low transfection efficiency High production cost	✗	[55,56]
	liposome-polycation-DNA nanoparticle	✓	✓	Fibrosarcoma	HT-1080 cells Xenograft tumors	High transfection efficiency Good biocompatibility	Complex structure and synthesis	✗	[57]
	lipid calcium phosphate nanoparticle	✓	✓	Non-small cell lung cancer	Xenograft tumors	High transfection efficiency Good biocompatibility Low toxicity	Complex structure and synthesis	✗	[58]
	exosomes	✗	✗	-	Mouse λ820 cells	High encapsulation efficiency Natural carriers Good biocompatibility Steady release profile	Lack of standardized techniques for isolation and purification	✗	[66]
	chitosan nanoparticles	✓	✓	Breast cancer	MCF-7 cells Xenograft tumors	Small particle size Good biocompatibility	Low transfection efficiency	✗	[67]
	poly(lactic-co-glycolic acid) nanocapsule	✗	✗	Neuroblastoma	-	High stability Biodegradability FDA-approved material Sustained release	Low transfection efficiency	✗	[68]
	siNozyme	✗	✗	melanoma	-	Biocompatibility Good bioavailability	Complex structure and synthesis	✗	[69]
	cell-penetrating peptide	✗	✓	melanoma	B16 cells Xenograft tumors	High transfection efficiency Low toxicity	The possible need for covalent conjugation Low cell specificity	✗	[70]
	multi-peptide complex	✗	✓	Breast cancer	MDA-MB-231	High transfection efficiency Low toxicity	Complex structure and synthesis	✗	[71]
	gold nanoparticles	✓	✓	Cervical cancer Breast cancer Lung cancer	HeLa cells MCF-7 cells Xenograft tumors A549 cells CMT/167 cells	Large surface area-high loading capacity Amenable to chemical manipulation	Toxicity	✗	[81,82,83,84,85,86,87]
	selenium nanoparticles	✓	✓	Glioblastoma	U251 tumor spheroids	Large surface area-high loading capacity Amenable to chemical manipulation	Toxicity	✗	[88]
	nano-graphene oxide	✓	✗	Breast cancer	MCF-7 cells MDA-MB-231 cells	High surface area to volume ratio Flexibility for cargo loading Amenable to functionalization	Adverse interactions with proteins Toxicity Immunogenicity	✗	[89]
DsiRNA	EnCore™ lipid nanoparticle	✗	✗	Advanced solid tumors Multiple myeloma Lymphoma	-Patients	High carrying capacity Good biocompatibility	Poor tumor penetration Unsatisfactory knockdown efficiency Expensive Labor intensive	✓	[91]
shRNA expression plasmid	cationic liposome	✗	✗	Breast cancer Liver cancer Colon cancer Nasopharyngeal cancer	MCF-7 cells Xenograft tumors HepG2 cells Colo320 cells CNE-2Z	High encapsulation efficiency Good biocompatibility Low toxicity Weak immunogenicity	Low transfection efficiency High production cost	✗	[93,94,95,96]
	polyglycidal methacrylate nanoparticle	✓	✗	Breast cancer Colorectal cancer	-	Low toxicity at high concentrations	Low transfection efficiency Complex structure and synthesis	✗	[99]
